# Art, science and the mental health we need in a pandemic

**DOI:** 10.1590/2237-6089-2020-0068

**Published:** 2020-10-08

**Authors:** Giovanni Abrahão Salum, Carlos Eduardo, Sara Evans-Lacko, Marcelo Pio de Almeida Fleck, Fernanda Lucia Capitanio Baeza

**Affiliations:** 1 Unidade Centro de Atenção Psicossocial, Serviço de Psiquiatria Hospital de Clínicas de Porto Alegre Porto AlegreRS Brazil Unidade Centro de Atenção Psicossocial, Serviço de Psiquiatria , Hospital de Clínicas de Porto Alegre (HCPA), Porto Alegre , RS , Brazil .; 2 Departamento de Psiquiatria e Medicina Legal Universidade Federal do Rio Grande do Sul Porto AlegreRS Brazil Departamento de Psiquiatria e Medicina Legal , Universidade Federal do Rio Grande do Sul (UFRGS), Porto Alegre , RS , Brazil .; 3 Faculdade de Medicina UFRGS Porto AlegreRS Brazil Faculdade de Medicina , UFRGS , Porto Alegre , RS , Brazil .; 3 Care Policy and Evaluation Centre London School of Economics and Political Science London United Kingdom Care Policy and Evaluation Centre , London School of Economics and Political Science , London , United Kingdom .

Mental health is a discipline that aims to provide the best possible care for people in need of mental health support. It is an extremely complex discipline fueled by tensions such as science vs. art, objectivity vs. subjectivity, statistics vs. stories, medication vs. psychotherapy, brain vs. mind, among others.

All these tensions are real. They are present in each clinical encounter between mental health professionals and patients: whether to rely on the results of a rating scale or an unstructured patient interview; to choose between a psychological or a pharmacological treatment; or to choose to perform a brain scan or explore art work a patient has made. Some may feel that they have to make a choice between the right or left side of those tensions, i.e., to choose between being a “soulless brain professional” or a “humanist scienceless practitioner.”

Here we argue that either form of reductionism ignores the complexity of patients’ needs. The mental health we need is plural. We need science and art; objectivity and subjectivity; statistics and stories; medication and psychotherapies; both brains and minds. Of course, the complexity (and beauty) of this discipline relies precisely on adapting and choosing the best perspective for each clinical situation. ^1^ Pluralists called this “changing the glasses” or “shifting gears”, ^2^ i.e., adapting perspectives to the specific needs and context of each patient.

In the face of coronavirus disease 2019 (COVID-19), we believe this form of thinking is even more important. Patients with severe mental illness need us to go beyond this type of reductionism. They need us to adapt our practices collectively to apply the best evidence to their care, but also be open to new types of care which address these unprecedented circumstances. And this requires both science and art, innovation and creativity.

We recently published a paper that reports on the “statistics” of our service during the COVID-19 lockdown. ^3^ We shared a spreadsheet tool for facilitating telemonitoring and tracking patients that are at risk of food insecurity (https://figshare.com/s/826f200d872e35ea67f1). Now, it is time to also publish the “art” that captures the services we need at this time.

**Figure 1:**
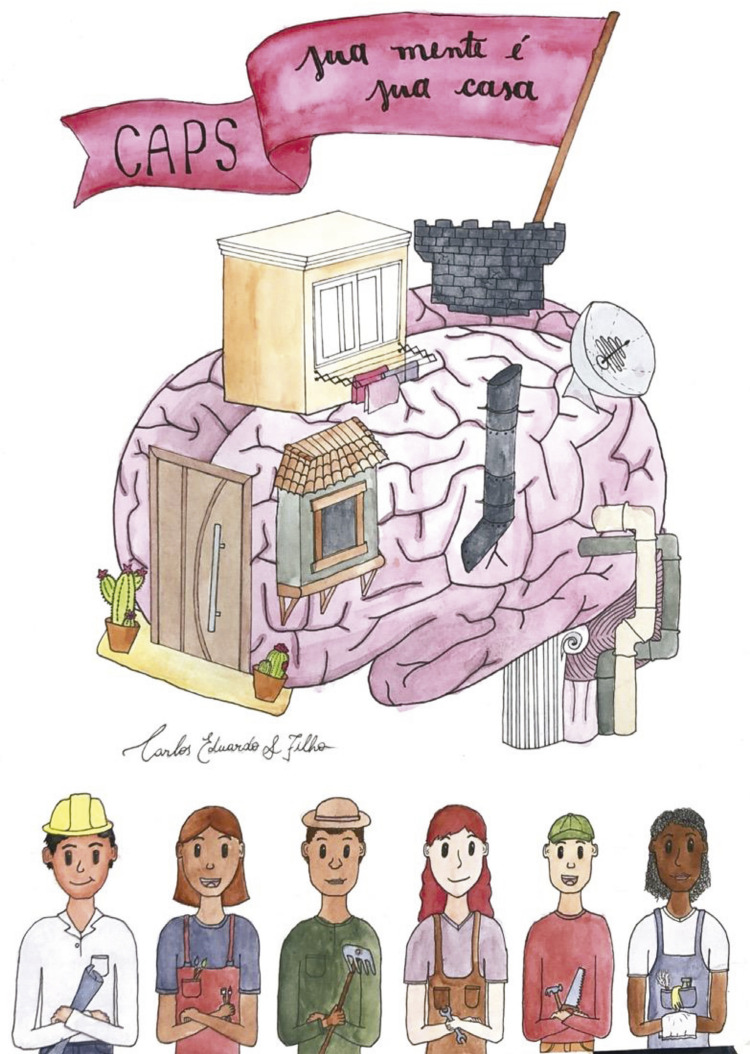
Pluralism of the Community Psychosocial Center captured by artist and medical student Carlos Eduardo Filho

Artist Carlos Eduardo Filho, a student attending the 7th semester of medical school at Universidade Federal do Rio Grande do Sul, southern Brazil, spent some time at our center and captured the pluralism of our practice (his artwork is shown in Figure 1 ). In these difficult times we are living, let us all embrace and look at what people with mental conditions need! This still is one of the most important debates of our discipline for providing the best possible care for patients.
